# *ASPM* and the Evolution of Cerebral Cortical Size in a Community of New World Monkeys

**DOI:** 10.1371/journal.pone.0044928

**Published:** 2012-09-27

**Authors:** Fernando A. Villanea, George H. Perry, Gustavo A. Gutiérrez-Espeleta, Nathaniel J. Dominy

**Affiliations:** 1 School of Biological Sciences, Washington State University, Pullman, Washington, United States of America; 2 Department of Anthropology, University of California Santa Cruz, Santa Cruz, California, United States of America; 3 Escuela de Biología, Universidad de Costa Rica, San José, Costa Rica; 4 Department of Anthropology, Pennsylvania State University, University Park, Pennsylvania, United States of America; 5 Department of Anthropology, Dartmouth College, Hanover, New Hampshire, United States of America; 6 Department of Biological Sciences, Dartmouth College, Hanover, New Hampshire, United States of America; University of Massachusetts, United States of America

## Abstract

The *ASPM* (abnormal spindle-like microcephaly associated) gene has been proposed as a major determinant of cerebral cortical size among primates, including humans. Yet the specific functions of *ASPM* and its connection to human intelligence remain controversial. This debate is limited in part by a taxonomic focus on Old World monkeys and apes. Here we expand the comparative context of *ASPM* sequence analyses with a study of New World monkeys, a radiation of primates in which enlarged brain size has evolved in parallel in spider monkeys (genus *Ateles*) and capuchins (genus *Cebus*). The primate community of Costa Rica is perhaps a model system because it allows for independent pairwise comparisons of smaller- and larger-brained species within two taxonomic families. Accordingly, we analyzed the complete sequence of exon 18 of *ASPM* in *Ateles geoffroyi*, *Alouatta palliata*, *Cebus capucinus*, and *Saimiri oerstedii*. As the analysis of multiple species in a genus improves phylogenetic reconstruction, we also analyzed eleven published sequences from other New World monkeys. Our exon-wide, lineage-specific analysis of eleven genera and the ratio of rates of nonsynonymous to synonymous substitutions (d*_N_*/d*_S_*) on *ASPM* revealed no detectable evidence for positive selection in the lineages leading to *Ateles* or *Cebus,* as indicated by d*_N_*/d*_S_* ratios of <1.0 (0.6502 and 0.4268, respectively). Our results suggest that a multitude of interacting genes have driven the evolution of larger brains among primates, with different genes involved in this process in different encephalized lineages, or at least with evidence for positive selection not readily apparent for the same genes in all lineages. The primate community of Costa Rica may serve as a model system for future studies that aim to elucidate the molecular mechanisms underlying cognitive capacity and cortical size.

## Introduction

A disproportionately large cerebral cortex is a hallmark of human evolution. It facilitates increased information processing and thus our perceived high level of intelligence and rapid rate of cultural innovation; for instance, our capacity for tool-making, social intelligence, and language [1]. As a result, considerable attention has long been focused on how and why a relatively large cerebral cortex was favored in some primate lineages. In recent years a particular emphasis has been paid to the identification of genetic variants that correlate with increased cortical size [2]. In this vein, the *ASPM* (abnormal spindle-like microcephaly associated) gene has been proposed as a major determinant of cerebral cortical size among primates [3], [4].

The *ASPM* gene encodes a 10,434-bp-long coding sequence, in 28 exons, and spans 65 kb of genomic DNA. Exon 3 spans approximately 1500 bp while exon 18 spans approximately 4700 bp. The remaining exons of the *ASPM* gene span less than 200 bp each. The *ASPM* gene encodes a protein that is widely conserved between primates. The ASPM protein contains four distinguishable regions: a putative N-terminal microtubule-binding domain, a calponin-homology domain, an IQ repeat domain containing multiple IQ repeats (calmodulin-binding motifs), and a C-terminal region [5]. More than half of the primate ASPM protein consists of repeated calmodulin-binding IQ domains. The major ASPM transcript contains 81 IQ domains, which are organized into IQ calmodulin-binding motifs comprised by 20–25 amino acids. The majority of these repeats are encoded in Exon 18.

Calmodulin binding to IQ motifs induces a conformational change in proteins that regulate the binding of actin to the aminoterminal CH domains. It has been proposed that changes in ASPM induce changes in the orientation of the mitotic spindle of neuroblasts, which induces symmetric mitosis generating two progenitor cells; as opposed to one progenitor cell and one postmitotic neuron, typical of asymmetric mitosis. The additional rounds of symmetric duplication cause an exponential expansion of the progenitor pool. Control of this proliferative symmetry can cause dramatic alterations in cerebral cortical size, and so changes in ASPM could regulate cortical size by making subtle changes in spindle orientation [6]. Given the proposed role of ASPM in regulating divisions of neuronal progenitors, both the number of repeats and the particular amino acid substitutions in the IQ repeats may be strongly related to brain evolution [5].

Such claims have fueled further research on the biology and function of *ASPM* [7], [8]. For example, Kouprina et al. [6] found that *ASPM* is expressed in numerous proliferating tissues outside the cerebral cortex, suggesting it has functions apart from neuroblast replication. Other authors linked *ASPM* to more general mechanisms such as ciliary function and spermatogenesis rather than neural development, further confusing the functional significance of *ASPM* during human evolution [9], [10], [11]. More controversially, a haplotype of *ASPM* was linked to recent and ongoing selective sweeps among populations of modern humans [12], [13], although these conclusions were subsequently challenged [14], [15] and links between *ASPM* genetic variants and human intelligence have been refuted [16]. Together, these studies improve our understanding of *ASPM* function, but do little to resolve why *ASPM* evinces a purported signature of positive selection in certain primate lineages.

In general, ties between *ASPM* and brain size evolution have been based on Old World monkeys, apes, and humans [3], [4], [5], [17]. Such an emphasis on catarrhine primates is logical given the level of shared ancestry, but it offers limited comparative context or independent power. To address this taxonomic void, Ali and Meier [18] and Montgomery et al. [19] included New World monkeys in their tests for positive correlations between *ASPM* variation and brain size across primates using codon-specific maximum likelihood tests of selection. Such an approach aimed to detect mutations in specific sites that might have impacted the function of the ASPM protein in specific lineages in ways that might have positively affected the fitness of an individual that carried the mutation (such that the mutation would have swept to fixation rapidly).

Ali and Meier [18] reported signatures of positive selection associated with relatively larger cerebral cortical volumes in nine primate lineages, including humans, chimpanzees, and a family of New World monkeys, the Atelidae. In contrast, the expanded analysis of Montgomery et al. [19] found little support for such specific episodes of adaptive evolution in any given lineage. Instead, they reported a positive relation between molecular evolution in *ASPM* and an increase in neonatal brain size across 21 anthropoid genera based on a fraction (2.3%) of the codon sites predicted to present a significant increase in non-synonymous substitutions over synonymous substitutions. Accordingly, Montgomery et al. [19] proposed a deeper evolutionary history of *ASPM*, and suggested that it might be responsible for independent increases in brain size in all major clades of primates, including New World monkeys.

Montgomery et al.’s [19] analysis of 10 species of New World (platyrrhine) monkey is instructive for highlighting the complexity of *ASPM* evolution within primates. However, if positive selection of *ASPM* is related to an ancestral increase in brain size, a comparative analysis requires the inclusion of multiple related species within genera, which allows for proper reconstruction of the ancestral states of *ASPM* sequences. Here we expand on these earlier findings by incorporating platyrrhine genera from all previous studies and adding four species from the primate community of Costa Rica, for a total of 16 species. Our analysis not only allows for a more complete treatment of the families Atelidae and Cebidae, but it also highlights the practical value of focusing on communities as model systems for studying the evolution of cerebral cortical size.

### Primates of Costa Rica

Although a sparse fossil record obscures the origins of New World monkeys, all present evidence suggests a trans-Atlantic dispersal event ca. 25–40 million years ago [20]. The subsequent adaptive radiation is remarkable for its variation in brain size, diet, and social behavior. Accordingly, New World monkeys provide fertile ground for exploring the underlying genetic mechanisms of large brain size. In this regard, the primate community of Costa Rica is a model system. It features sympatric species in the family Atelidae - the mantled howling monkey (*Alouatta palliata*) and the black-handed spider monkey (*Ateles geoffroyi*). The two species are comparable in body size, yet the brain of *A. geoffroyi* is nearly twofold larger (Table 1). Similarly, two species in the family Cebidae coexist in some habitats - the white-faced capuchin (*Cebus capucinus*) and the squirrel monkey (*Saimiri oerstedii*). The relative brain size of *Cebus* is among the largest of any nonhuman primate and 10–20% larger than that of *Saimiri* (Table 1) [19], [21]. It also has a complicated structure. The cerebral cortex of *Cebus* shares with *Ateles* the most complex pattern of fissures among the platyrrhine primates [22]. Compellingly, such parallel increases in brain size and complexity support an expanded coterie of cognitive abilities.

**Table 1:** Brain mass and volumes of genera in the families Atelidae and Cebidae [modified from 19].

	Adult				
Genus	Body mass (g)	Brain mass (mg)	Brain volume (mm3)	Neocortex volume (mm3)	Telencephalon volume (mm3)
***Alouatta***	6400.0	52000.0	49009.0	31660.0	37388.0
***Ateles***	8000.0	108000.0	101034.0	70856.0	79946.0
***Cebus***	3100.0	71000.0	66939.0	46429.0	52113.0
***Saimiri***	660.0	24000.0	22572.0	15541.0	17635.0
	**Neonate**				
**Genus**	**Body mass (g)**	**Brain mass (mg)**			
***Alouatta***	363.9	30800.0			
***Ateles***	512.0	63950.0			
***Cebus***	232.9	33650.0			
***Saimiri***	153.5	15240.0			

### Cognitive Ecology and Brain Size

The tropical forests of Costa Rica are highly dynamic fluctuating systems that require complex foraging strategies to meet nutritional needs over all phases of an annual cycle. Despite similar body sizes, *Ateles* and *Alouatta* have evolved contrasting dietary behaviors. In northwestern Costa Rica, *Alouatta palliata* can devote up to 77% of its monthly foraging time to eating leaves [23], whereas *Ateles geoffroyi* shows a strong predilection for fruit and flowers (up to 78% and 10% of foraging time, respectively) [24]. Compared to the ubiquity of leaves, fruit and flowers are scattered spatially and temporarily; thus, for *Ateles*, the selective advantages associated with remembering the location and availability of such patchy resources has been tied to the evolution of its relatively large brain [25]. In fact, the memory skills of *Ateles* are reported to exceed those of squirrel monkeys and macaques under experimental conditions [26].

*Cebus* and *Saimiri* also share similar body sizes and morphologies, and often forage sympatrically on the same substrates [22]. However, *Cebus* and *Saimiri* differ considerably in the extent to which they acquire resources manually [27]. Whereas *Saimiri* tends to glean invertebrates from exposed surfaces, *Cebus* spends ca. 50% of its day searching for and extracting embedded foods [22]. Such extractive foraging behavior is expected to favor greater manual dexterity and a high degree of sensorimotor intelligence [28], [29]. Sensorimotor intelligence, in turn, is hypothesized to drive technical innovation; indeed, *Cebus* manufactures the greatest variety of tools and uses them more often than all nonhuman primates excepting chimpanzees [30]–[32]. For *Cebus*, the cognitive demands and selective advantages associated with conceptualizing unseen foods and the use of tools to extract them was possibly a major contributing factor to the evolution of its large brain [33].

Given the ecological and cognitive challenges faced by *Ateles* and *Cebus*, i.e. the need to remember scarce resources or imagine and extract concealed ones, it is worth testing whether there has been convergent adaptive evolution in these lineages of the same genes believed to be responsible for human cortex size and cognitive ability. More specifically, the pair-wise coexistence of large- and small-brained atelid and cebid primates in Costa Rica provides a model system for testing molecular hypothesis focused on the evolution of brain size. In this study we compared the complete sequence of exon 18 in *ASPM* across eleven platyrrhine genera, including multiple species of large-brained *Ateles* and *Cebus*, and small-brained *Alouatta* and *Saimiri*, and studied patterns of natural selection on this gene coding sequence using both an exon-wide lineage-specific analysis similar to Evans et al. [4] and codon-specific analyses across taxa similar to those of Ali and Meier [18] and Montgomery et al. [19]. Exon 18 alone represents 45% of the *ASPM* gene and has been shown to code for most of the critically functioning region of the ASPM protein; the Cadmodulin-binding domain, consisting of repetitive IQ domains. Early d*_N_*/d*_S_* analyses of anthropoid primates indicated different rates of evolution along the ASPM protein, where rapidly evolving residues were mainly concentrated in the IQ repeat domain, coded in its majority by Exon 18, making it the prime region to consider in studies of natural selection [6], [10], [18], [19], [21].

## Results and Discussion

**Figure 1:**
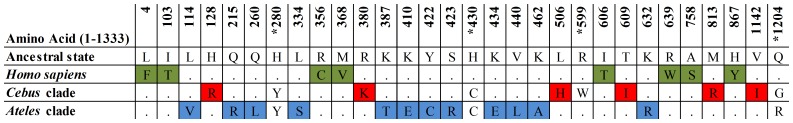
Amino acid replacements of ASPM exon 18 specific to *Homo*, *Cebus* and *Ateles*. Positions denoted by a star (*) are predicted to be under positive selection by the Bayes Empirical Bayes associated test (p<0.05).

**Figure 2:**
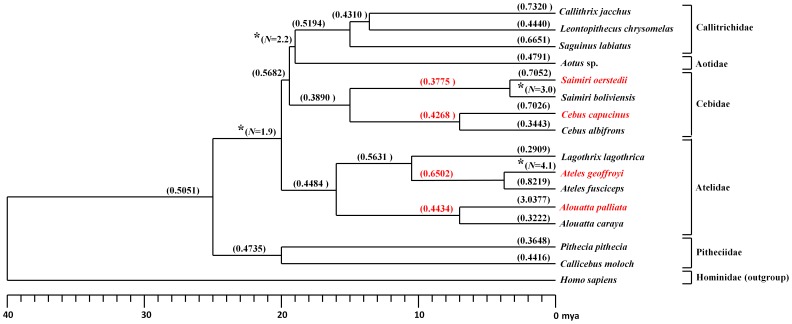
Exon-wide lineage-specific test of neutrality: d*_N_*/d*_S_* ratios for eleven new world monkey species. Genera with novel added species are marked in red, d*_N_*/d*_S_* values for the ancestral branch of those genera are also marked in red. Ratios significantly greater than 1 suggest positive selection while ratios significantly less than 1 are consistent with purifying selection on nonsynonymous mutations. Ratios non-significantly different from 1 are consistent with neutrality. Asterisks represent a missing value; between these lineages there are no synonymous substitutions (d*_S_* = 0) and thus PAML is unable to properly estimate d*_N_*/d*_S_*. In these cases, the number of nonsynonymous substitutions (*N*) is reported.

Previously, *ASPM* was reported to be under positive selection in the lineages leading to humans and other primates with relatively increased cortex size. In particular, a human-chimpanzee comparison revealed repeated selection on multiple sites along the *ASPM* gene [4]. Here we focused on two genera of New World monkey, *Ateles* and *Cebus*, which evolved increased cortical sizes independently within their taxonomic families. In contrast to their shared increased cortical sizes, amino acid replacements specific to humans, *Cebus* and *Ateles* found in exon 18 of the ASPM protein do not overlap, underlying the independent evolution of this region in these species of interest (fig. 1). Lineage-specific analyses of exon 18 sequences of the *ASPM* gene resulted in d*_N_*/d*_S_* ratios of 0.6502 for the *Ateles* lineage and 0.4268 for the *Cebus* lineage (fig. 2). Such values (<1) are consistent with functional constraint on amino acid sequences, rather than positive selection. Our exon-wide lineage-specific analysis of the ratio of the rates of nonsynonymous (amino acid-changing, potentially functional) to synonymous (presumably neutral) substitution (d*_N_*/d*_S_*) on *ASPM* thus revealed no detectable evidence for positive selection. It should be noted that the only branch to present a positive d*_N_*/d*_S_* ratio, was that of *Alouatta palliata* (3.0377, fig. 2), however, this result was driven by a small number of estimated substitutions (*N* = 8.9, *S* = 1.0), and was not significantly different from a model of neutral evolution (p = 0.235).

Although our exon-wide lineage-specific results within the Atelidae stand in contrast with those of Ali and Meier [18], we also followed their analytical approach with codon-specific models of selection. This is accomplished by allowing d*_N_*/d*_S_* to vary across codon positions as well as across lineages, and then recording the differences in the distribution of positively selected changes between “foreground” branches (*Cebus* and *Ateles* branches) and the rest of the phylogenetic tree. Our results from this test also failed to reject a model of neutral evolution in the *Ateles* and *Cebus* lineages (p = 0.491). The discrepancy in results may be due to the addition of an extra species within the genus *Ateles*, allowing for better phylogenetic reconstruction.

Finally, following the methodology of Montgomery et al. [19], we also implemented a branch-site model, which allows ω to vary across amino acid sites, but not across lineages. This model was used for identifying amino acid sites deviating from neutrality, which could be indicative of gradual positive selection across the entire phylogeny, as opposed to simply from the ancestral primate “root” to each terminal branch “tip”. Our site test of gradual selection failed to reject a model of neutral evolution within platyrrhine monkeys (p = 0.998). It should be noted that Montgomery et al. [19] only rejected a model of neutral evolution using this test when all anthropoid primates were included, but they did not detect a signature of overall positive selection specifically within New World monkeys (as reported by a d*_N_*/d*_S_* value of 0.479 [19]), which is consistent with our results. Montgomery et al. [19] also predicted 2.3% of all amino acid sites in their sequences to have been under positive selection across all primates, based on the Bayes Empirical Bayes (BEB) analysis associated with the method. Our own BEB analysis, specific for New World monkeys, predicted four amino acid sites presenting significant deviations from neutrality (ω>1, p<0.05, fig. 1). Those sites however, only represent a small portion (0.3%) of all amino acid sites, and our overall model was not significantly different from neutrality. Therefore, these few positive results may not be biologically meaningful. We interpret these results to indicate no evidence of positive selection across the platyrrhines.

### Conclusion

In contrast to the results of Ali and Meier [18], we found a relatively gradual tempo of neutral evolution of *ASPM* in the Atelidae, and no evidence of strong positive selection in any other lineage of New World monkeys. We also found no evidence of gradual positive selection over the platyrrhine clade, despite evidence for independent cerebral enlargement in the lineages leading to *Ateles* and *Cebus*. This finding is somewhat contradictory to Montgomery et al.’s [19] conclusion of shared genetic bases for encephalization across anthropoid primates (which includes platyrrhine monkeys). However, it should be noted that both Ali and Meier [18] and Montgomery et al. [19] included exon 3 of *ASPM* into their analysis, so it is possible that our results are consistent with positive selection occurring on exon 3. Because changes in exon 18 are most likely to affect neural cell duplication, we expected positive selection associated with brain size increase to occur in this region. It is possible that positive selection of *ASPM* is associated with processes other than brain development, at least in New World monkeys, thus explaining the inconsistencies between our results and previously published results.

We note that it is also possible that our tests had insufficient power to detect evidence of positive selection on any specific changes in *ASPM* exon 18, even if such changes did in fact partly account for the large differences in brain anatomy among New World monkeys. It is also plausible that changes in other *ASPM* exons that we did not study, or in *ASPM* regulatory regions, may harbor evidence of positive selection in these taxa. Of course, brain development and higher cognitive skills are almost certainly affected by a multitude of interacting genes, such that convergent phenotypic features may be achieved through different modifications on separate lineages.

The primate community of Costa Rica, featuring coexisting small- and large-brained species with remarkable behavioral diversity, has the potential to be an instructive model system for testing hypotheses relevant to human evolution. By contrasting candidate genes such as *ASPM* with species under similar selective pressures, one might be able to provide insight into human neurogenetics. Species such as *Cebus* and *Ateles* offer independent examples of encephalization and relatively increased intelligence among primates.

## Materials and Methods

### Ethics Statement

We affirm that our research methods were approved by all relevant government agencies and the Chancellor’s Animal Research Committee of the University of California, Santa Cruz (approval no. 0906). UC-Santa Cruz was the institutional affiliation of the corresponding author at the time of the research. Samples were collected with the approval of Costa Rica’s Ministerio de Ambiente y Energía (permit no. 069-2001 OFAU). Buccal swabs containing DNA were exported from Costa Rica (Ministerio de Agricultura y Ganaderia permiso fitosanitario no. 331) and imported into the USA (CITES permit no. 06US130146/9).

### Samples and DNA Analysis

One of the authors (G.A.G.-E.) tranquilized wild individuals [34] of four species (*Alouatta palliata*, *Ateles geoffroyi*, *Cebus capucinus*, *Saimiri oerstedii*) in the Pacific region of Costa Rica (Ministerio de Ambiente y Energía permit no. 069-2001 OFAU). Buccal swabs containing DNA were exported from Costa Rica (Ministerio de Agricultura y Ganaderia permiso fitosanitario no. 331) and imported into the USA (CITES permit no. 06US130146/9). The DNA was extracted using a standard Qiagen DNeasy blood and tissue extraction kit. This protocol was reviewed and approved by the Chancellor’s Animal Research Committee of the University of California, Santa Cruz (approval no. 0906).

Standard PCR conditions were used to amplify *ASPM*. Primers were designed specifically for exon 18 (Table S1). Invitrogen Standard TOPO cloning was used with some amplicons (Vector primers available in Table S1). In these cases, three clones were sequenced per amplicon and a consensus of all three was used to rule out replication errors. All sequencing was done using standard dye terminator chemistry on PCR products at UC Berkeley sequencing facilities with an Applied Biosystems 96 capillary-3730xi genetic analyzer.

### Sequence Analysis

Comparative exon 18 sequence for *Homo sapiens* was taken directly from the UCSC Genome Browser (http://www.genome.ucsc.edu/, NCBI36/hg18, March 2006). All new sequences were deposited into GenBank with the following accession numbers: *Alouatta palliata* [GQ221757], *Ateles geoffroyi* [GQ221758], *Cebus capucinus* [GQ221759], *Saimiri oerstedii* [GQ221760]. Sequences for the remaining species were acquired from GenBank: *Saimiri boliviensis* [AY485419]; *Aotus* sp. [AY485422; [4]]; *Saguinus labiatus* [AY497015; [5]]; *Ateles fusciceps* [FJ013122]; *Lagothrix lagotricha* [FJ013130; [18]]; *Callithrix jacchus* [HQ540102]; *Cebus albifrons* [HQ540103]; *Alouatta caraya* [HQ540104]; *Leontopithecus chrysomelas* [HQ540105]; *Callicebus moloch* [HQ540106]; *Pithecia pithecia* [HQ540107; [19]].

Sequence alignment was performed in Bioedit [35]. A species tree was assembled based on the primate phylogeny published in Perelman et al. [36]. Next, MEGA 4.0 beta 4020 [37] and DAMBE [38] were used to prepare input data for PAML 4 [39]. The d*_N_*/d*_S_* ratios were estimated in PAML using model 1. In this model, ω is the average d*_N_*/d*_S_* within lineages with independent ω’s between lineages. In the absence of synonymous substitutions, the d*_N_*/d*_S_* ratio cannot be calculated; as a result, some of our branches lack a d*_N_*/d*_S_* ratio (fig. 1). Because synonymous substitutions were often absent between closely related species within genera, (d*_S_* = 0), PAML is unable to properly estimate d*_N_*/d*_S_*. In these cases d*_N_* was reported individually. To test if the high value of d*_N_*/d*_S_* in *Alouatta palliata* is significantly different from neutrality, a specific model in which ω evolves at a different rate for this branch was tested against a neutral model using a likelihood ratio test.

As suggested by Ali and Meier [18], a branch-site test of neutrality (to potentially identify positive selection) was performed as recommended by Yang [39] using model 2 in PAML. This branch site model was specifically designed to test for episodes of positive selection (as deviations from neutrality) acting on a small number of branches [40], by using a maximum likelihood approach to detect codon-specific positive selection and allowing ω to vary across codon positions as well as across priori-assigned foreground and background lineages. In this case, *Ateles* and *Cebus* branches were selected as foreground branches based on behavioral and morphological observations. The cut-off for statistical significance was assigned at the 0.05 level of probability by testing twice the difference of the log-likelihood (2Δl) output from PAML compared against a chi-square distribution (d.f. = 1).

Following the recommendations of Montgomery et al. [19] we adopted a branch site model, which allows ω to vary across sites, but not across lineages, and tests for deviations from neutrality across a phylogeny. Site models M1a (null model of neutral evolution) and M2a (alternative model of positive selection) were compared using a likelihood ratio test statistic with a cut-off for statistical significance assigned at the 0.05 level of probability by testing twice the difference of the log-likelihood (2Δl) output from PAML compared against a chi-square distribution (d.f. = 2) [19], [39]. In addition, this model utilizes Yang’s Bayes Empirical Bayes (BEB) model to predict amino acid sites where ω>1 at the 0.05 and 0.01 levels of probability. Because some of the results in Montgomery et al. [19] were only significant when the callitrichids were removed from their analysis, we repeated all analysis after removing *Saguinus labiatus*, *Callithrix jacchus* and *Leontopithecus chrysomelas* from our sample. The removal of this family led to no differences in results for the remaining species.

## Supporting Information

Table S1 **ASPM Exon 18 primers and Invitrogen TOPO cloning vector primers.**
(XLS)

File S1 **Abstract (Spanish).**
(DOC)
