# Supplementary material for: ASPM and the Evolution of Cerebral Cortical Size in a Community of New World Monkeys
Source: PLoS One. 2012 Sep 27;7(9):e44928. doi: 10.1371/journal.pone.0044928 (PMC3459963; doi:10.1371/journal.pone.0044928)
Supplement: File S1 — Abstract (Spanish). (DOC) [file pone.0044928.s002.doc]

## Abstract (Spanish)

El gen *ASPM* (abnormal spindle-like microcephaly associated) ha sido propuesto como un factor determinante del tamaño cerebral en primates, incluyendo humanos. Sin embargo, la función específica de *ASPM* y su posible conexión con la inteligencia humana es aún controversial. En parte, este debate se debe a que el estudio de *ASPM* se ha limitado a simios y monos del viejo mundo. En este articulo, expandimos el contexto comparativo de este gen para incluir monos del nuevo mundo, una radiación adaptativa de primates en la que el tamaño cerebral ha evolucionado en paralelo en monos araña (genero *Ateles*) y monos capuchinos (genero *Cebus*). La comunidad de primates de Costa Rica es un sistema modelo que permite comparar especies de mayor y menor tamaño cerebral dentro de la misma familia taxonómica. Con ese fin, secuenciamos el exon 18 de *ASPM* en *Ateles geoffroyi*, *Alouatta palliata*, *Cebus capucinus*, y *Saimiri oerstedii*. El análisis de varias especies dentro del mismo género permite una mejor reconstrucción filogenética, y con ese fin, añadimos once secuencias publicadas en la literatura de otros monos del nuevo mundo. Nuestro análisis del exon, específico para cada rama, y análisis de substituciones no-sinónimas a sinónimas (d*_N_*/d*_S_*) de *ASPM* no han revelado ninguna evidencia de selección positiva en las ramas que llevan a *Ateles* o *Cebus*, como indican valores menores a uno 0.6502 y 0.4268, respectivamente). Nuestros resultados sugieren que la interacción de varios genes fueron responsables del aumento de tamaño cerebral en primates, a al menos la evidencia de selección positiva no está presente en todos los grupos de primates. La comunidad de primates de Costa Rica puede servir como un sistema modelo para el futuro estudio de los mecanismos moleculares responsables del tamaño cerebral y la capacidad cognoscitiva.
